# Real-world treatment patterns and survival outcomes in extensive-stage small cell lung cancer across first-line induction and maintenance therapy

**DOI:** 10.1093/oncolo/oyag434

**Published:** 2026-11-04

**Authors:** Yi-Shao (Justus) Liu, Parth A Desai, Humera Khurshid, Anne C Chiang, Christine L Hann, Melissa L Johnson, Erik S Anderson, Rani Najdi, Jessie Wang, Xerxes Pundole

**Affiliations:** Amgen Inc, Thousand Oaks, CA 91320, United States; Department of Hematology & Medical Oncology, Fox Chase Cancer Center, Temple Health, Philadelphia, PA 19140, United States; Brown University School of Medicine-Rhode Island Hospital, Providence, RI 02903, United States; Yale Cancer Center, Yale University School of Medicine, New Haven, CT 06520, United States; Johns Hopkins Sidney Kimmel Comprehensive Cancer Center, Baltimore, MD 21287, United States; Sarah Cannon Research Institute, Nashville, TN 37203, United States; Amgen Inc, Thousand Oaks, CA 91320, United States; Amgen Inc, Thousand Oaks, CA 91320, United States; Amgen Inc, Thousand Oaks, CA 91320, United States; Amgen Inc, Thousand Oaks, CA 91320, United States

**Keywords:** small cell lung cancer, immune checkpoint inhibitors, real-world evidence, maintenance treatment

## Abstract

**Background:**

Platinum-etoposide (PE) chemotherapy combined with a programmed cell death ligand 1 (PD-L1) inhibitor is the current first-line (1L) standard of care for extensive-stage small cell lung cancer (ES-SCLC). Real-world evidence describing outcomes across induction and maintenance phases remains limited.

**Patients and Methods:**

This descriptive retrospective observational study used the US-based, electronic health record-derived deidentified Flatiron Health Research Database (January 2013-December 2024). Adults with pathologically confirmed ES-SCLC at initial diagnosis initiating 1L therapy were included. Patients were categorized by 1L regimen: PE plus a PD-L1 inhibitor (chemo-immunotherapy), PE alone, or other regimens. Maintenance was defined as at least 2 consecutive doses of post-induction PD-L1 inhibitor monotherapy. Real-world overall survival (rwOS) and progression-free survival (rwPFS) were estimated using the Kaplan-Meier method.

**Results:**

Among 5863 1L patients, 2157 (36.8%) received chemo-immunotherapy, of whom ∼60% (1284/2157) received maintenance (atezolizumab, 85.7%; durvalumab 14.3%); 3376 (57.6%) received PE, and 330 (5.6%) other regimens. Median rwOS and rwPFS from 1L initiation were 8.1 months (95% CI, 8.0-8.3) and 5.1 months (95% CI, 5.0-5.2), respectively. From 1L initiation, patients who subsequently met the maintenance definition had median rwOS of 12.2 months. From maintenance initiation, median rwOS was 8.7 months (95% CI, 8.0-9.8) with atezolizumab and 9.3 months (95% CI, 8.0-11.5) with durvalumab.

**Conclusion:**

In this large US real-world cohort, survival outcomes in 1L ES-SCLC patients were poor and appeared broadly comparable between atezolizumab and durvalumab. Only ∼60% of patients receiving chemo-immunotherapy subsequently received maintenance in routine clinical practice, underscoring substantial attrition before maintenance initiation.

Implications for practice Chemo-immunotherapy followed by PD-L1 inhibitor maintenance is the current first-line standard of care for ES-SCLC, yet real-world evidence describing outcomes across both the induction and maintenance phases remains limited. In this large US real-world study, only approximately 60% of patients receiving chemo-immunotherapy transitioned to maintenance therapy, highlighting substantial attrition before maintenance initiation. Among patients who received maintenance therapy, descriptive survival outcomes were broadly comparable between atezolizumab and durvalumab. These findings emphasize the need for strategies that improve outcomes earlier in the treatment course to ultimately extend long-term survival.

## Introduction

Small cell lung cancer (SCLC) accounts for approximately 15% of all lung cancers and is an aggressive malignancy characterized by rapid tumor growth, early dissemination, and frequent relapse.^1^ At diagnosis, an estimated 70%-80% of patients present with extensive-stage SCLC (ES-SCLC), while the remaining 20%-30% have limited-stage disease (LS-SCLC).^1^

The current first-line (1L) standard of care for ES-SCLC consists of platinum-etoposide (PE) chemotherapy administered in combination with a programmed cell death ligand 1 (PD-L1) inhibitor (chemo-immunotherapy) as induction therapy, followed by maintenance PD-L1 inhibitor with atezolizumab or durvalumab.^2^^,^^3^ In pivotal phase III trials, the addition of a PD-L1 inhibitor to PE chemotherapy resulted in statistically significant improvements in survival. In the IMpower 133 trial, atezolizumab plus PE improved median overall survival (OS) from 10.3 to 12.3 months.^4^ Similarly, durvalumab plus PE improved median OS from 10.5 to 12.9 months compared with PE alone in the CASPIAN trial.^5^ Despite these advances, ES-SCLC remains associated with poor long-term survival due to rapid resistance and modest survival benefits.^6^

Maintenance immunotherapy is now an established component of front-line ES-SCLC management.^7^ An exploratory analysis of IMpower133 demonstrated improved survival among patients who continued atezolizumab during the maintenance phase compared with those who received placebo (median OS from the start of maintenance therapy: 12.5 versus 8.4 months; hazard ratio [HR] = 0.59; 95% CI, 0.43-0.80).^8^ More recently, the phase III IMforte trial reported that lurbinectedin plus atezolizumab as a maintenance therapy prolonged median OS compared with atezolizumab monotherapy (13.2 vs 10.6 months; HR = 0.73; 95% CI, 0.57-0.95) in patients who had completed induction PE plus atezolizumab chemo-immunotherapy.^9^ These findings further highlight the clinical relevance of the maintenance phase as a potential window for therapeutic intensification.

In the real-world setting, a few studies have compared the effectiveness of PD-L1 inhibitors in ES-SCLC.^10–13^ Some analyses have reported numerically longer OS with durvalumab relative to atezolizumab.^11^^,^^12^ However, these studies were limited by small sample sizes and single-center designs. To our knowledge, few prior real-world investigations have evaluated outcomes specifically from the initiation of 1L maintenance therapy following completion of 1L induction chemo-immunotherapy.^10–13^ To address these gaps, we conducted this retrospective observational study to describe the evolving treatment landscape and to evaluate outcomes among patients with ES-SCLC across both the 1L induction and 1L maintenance settings.

## Methods

### Study design and patients

This retrospective, descriptive observational study included patients with ES-SCLC identified from the Flatiron Health Research Database between January 1, 2013 and December 31, 2023. Eligible patients were adults with pathologically confirmed ES-SCLC at initial diagnosis who initiated 1L systemic therapy during the study period. Patients initially diagnosed with LS-SCLC who subsequently developed recurrent or ES-SCLC were not included. ES-SCLC diagnosis was based on the SCLC-specific limited-stage/extensive-stage designation when available; if unavailable, only documented American Joint Committee on Cancer stage IV disease was mapped to ES-SCLC. The data cutoff date was December 31, 2024, allowing for a minimum of 12 months of potential follow-up after 1L therapy initiation. Patients were excluded if they lacked abstractable unstructured clinical documents, had a diagnosis of non-small cell lung cancer (NSCLC) on or before the date of SCLC diagnosis, or received a clinical trial drug or a PD-1 inhibitor (pembrolizumab, nivolumab, or cemiplimab) in the 1L setting.

### Data source

This study used the US-based electronic health records (EHR)-derived Flatiron Health Research Database.^14^ This database contains longitudinal, de-identified, patient-level data from approximately 280 cancer clinics representing ∼800 care sites nationwide. The database includes both structured data elements and unstructured clinical documents curated through technology-enabled abstraction by trained clinical abstractors. All data were de-identified and subject to safeguards to prevent re-identification and protect patient confidentiality.

### Treatment definitions and analysis cohorts

Lines of therapy were derived using a clinician-validated, rule-based algorithm incorporating both structured and unstructured EHR data. Based on the 1L induction therapy received, patients were categorized into 3 mutually exclusive cohorts: (1) PE plus PD-L1 inhibitors (chemo-immunotherapy), defined as atezolizumab or durvalumab in combination with etoposide and platinum-based chemotherapy, (2) PE chemotherapy alone, defined as etoposide plus platinum-based chemotherapy without PD-L1 inhibitors or other immunotherapies, and (3) Other regimens, defined as all other 1L systemic treatments not included in the above categories.

Among patients receiving 1L induction chemo-immunotherapy, receipt of at least 2 consecutive administrations of atezolizumab or durvalumab monotherapy after completion of induction, operationally defined the 1L maintenance (1LM) cohort. This 2-dose requirement was intended to identify an ongoing maintenance regimen and to distinguish it from an isolated post-induction PD-L1 inhibitor administration that might represent a delayed or catch-up induction dose. Patients with no qualifying post-induction administration or only 1 qualifying administration did not meet the operational maintenance definition and were classified as the non-maintenance cohort. Within the 1LM cohort, patients were further grouped by maintenance agent (atezolizumab or durvalumab). Baseline demographics (age, sex at birth, race, and smoking status) and clinical characteristics (Eastern Cooperative Oncology Group [ECOG] performance status, presence of brain or liver metastases) were summarized across cohorts and relevant subgroups. International Classification of Diseases (ICD) codes were used to identify brain metastasis (C79.3x or 198.3x) or liver metastases (C78.7x or 197.7x) from 30 days prior to initial ES-SCLC diagnosis through the respective index date.

### Outcomes

The primary outcomes were real-world overall survival (rwOS) and real-world progression-free survival (rwPFS). For analyses of the overall 1L cohort, the index date was defined as the initiation of 1L induction therapy. For patients in the 1LM cohort, outcomes were evaluated from the initiation of 1L induction therapy as well as anchored to the initiation of maintenance monotherapy.

rwOS was defined as the time from the index date to death from any cause. Patients without documented death by the data cutoff date were censored at the time of their last confirmed activity within the database. Death was captured using Flatiron Health’s composite mortality endpoint, which integrates structured EHR data, the Social Security Death Index, and obituary data, and has been validated across multiple types of cancers, including in patients with SCLC.^15^ rwPFS was defined as the time from the index date to the first real-world progression (rwP) event prior to initiation of second-line (2L) therapy or death from any cause. Patients who initiated second-line (2L) therapy, without an rwP event or death, were censored at the earlier of the last clinic note or 2L therapy initiation. rwP events were abstracted based on information gathered from various sources of evidence, including radiology reports, pathology findings, and clinician assessments documented in the medical record.^16^ The database captured the occurrence and timing of rwP but not its anatomical site or pattern and did not permit reliable distinction between progression at a preexisting versus new metastatic site or between single-site versus multi-site progression.

### Statistical analyses

All analyses were descriptive, and no formal hypothesis testing was performed. Treatment patterns of 1L induction therapy and maintenance therapy were summarized over time. Baseline characteristics were summarized for the overall 1L cohort and within subgroups defined by maintenance status (1LM vs non-maintenance) and type of maintenance PD-L1 inhibitor (atezolizumab vs durvalumab). Categorical variables were summarized using frequencies and percentages, and continuous variables using medians and ranges. Missing data were not imputed.

The Kaplan-Meier (KM) method was used to estimate rwOS and rwPFS. Median rwOS and rwPFS with 95% confidence intervals (CIs) were calculated, along with pre-specified survival probabilities at 6-, 12-, and 24-month timepoints. Prespecified subgroup analyses were conducted by age, sex at birth, race, ethnicity, ECOG performance status, smoking history, type of practice, use of PD-L1 inhibitor in 1L (for the 1L cohort), and presence of brain or liver metastases. Sensitivity analyses included: (1) rwOS analyses restricted to patients with ECOG performance status of 0 or 1 at index and receipt of at least 3 cycles of induction chemo-immunotherapy prior to maintenance initiation, to better align with eligibility criteria used in clinical trials; and (2) alternative rwPFS definition requiring confirmation of rwP by radiographic assessment, to better align with progression capture in clinical trials. Because analyses were descriptive and unadjusted, observed differences between treatment groups should not be interpreted as causal or comparative estimates of treatment effectiveness. All analyses were conducted using SAS version 9.4 (SAS Institute Inc., Cary, NC).

## Results

### Baseline characteristics

A total of 5863 patients with ES-SCLC who initiated 1L systemic therapy were included. In the 1L induction setting, 2157 (36.8%) received chemo-immunotherapy, 3376 (57.6%) received PE chemotherapy, and 330 (5.6%) received other therapy. Among those who received 1L induction chemo-immunotherapy, 1284 patients (59.6%) met the operational maintenance monotherapy definition and comprised the 1LM cohort. Of these, 1100 (85.7%) received atezolizumab and 184 (14.3%) received durvalumab (Figure 1).

**Figure 1.**
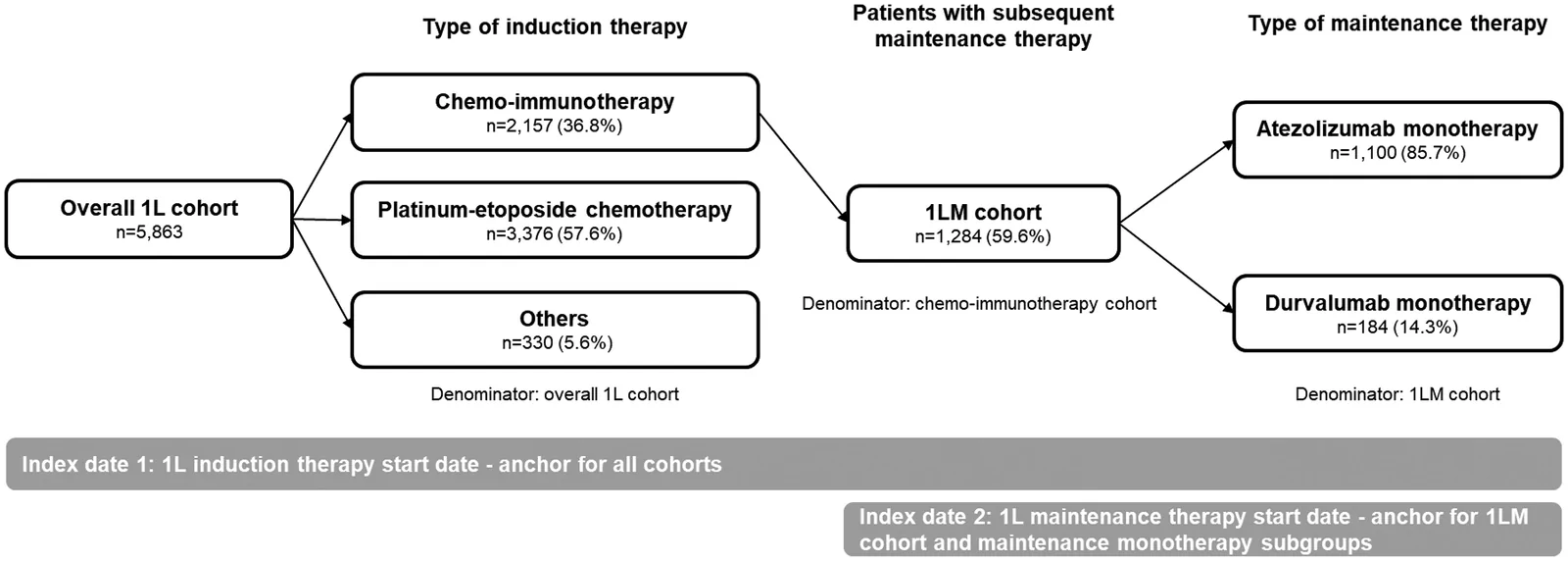
Patient cohort diagram. Time-to-event outcomes were estimated from prespecified index dates. Index date 1 was defined as initiation of 1L induction chemo-immunotherapy. For the 1LM cohort and atezolizumab/durvalumab monotherapy subgroups, analyses were additionally conducted from index date 2, defined as initiation of maintenance monotherapy. Abbreviations: 1L = first-line; 1LM = first-line maintenance.

Patient baseline and disease characteristics are provided in Table 1. In the overall 1L cohort (*N* = 5863), the median age at 1L induction therapy initiation was 68.0 years (range, 30-85) and 2904 (49.5%) patients were female. Most patients were White (73.6%) and had a history of smoking (97.9%). Overall, 4585 (78.2%) patients were treated in community practices. A little more than half 3193 (54.4%) had an ECOG performance score of 0 or 1 at initiation of 1L induction therapy (Table 1). Brain and liver metastases were identified in 630 (10.7%) and 919 (15.7%) patients, respectively, during the period from 30 days before the ES-SCLC diagnosis through initiation of 1L induction therapy.

**Table 1.** Baseline characteristics of patients with extensive-stage small cell lung cancer receiving 1L induction therapy.^a^

	Overall 1L cohort (*N* = 5863)	1L induction therapy		
		Chemo-immunotherapy (*N* = 2157)	Chemotherapy (*N* = 3376)	Others^b^ (*N* = 330)
**Median age at index date, years (range)**	68 (30-85)	68 (30-85)	68 (34-85)	67 (38-85)
**Age ≥65 years, *n* (%)**	3715 (63.4)	1416 (65.6)	2106 (62.4)	193 (58.5)
**Female, *n* (%)**	2904 (49.5)	1098 (50.9)	1637 (48.5)	169 (51.2)
**Race, *n* (%)**				
** Asian**	46 (0.8)	19 (0.9)	25 (0.7)	2 (0.6)
** Black or African American**	399 (6.8)	151 (7.0)	225 (6.7)	23 (7.0)
** White**	4313 (73.6)	1527 (70.8)	2542 (75.3)	244 (73.9)
** Other race**	432 (7.4)	140 (6.5)	271 (8.1)	21 (6.4)
** Missing/unknown**	673 (11.5)	320 (14.8)	313 (9.3)	40 (12.1)
**Ethnicity, *n* (%)**				
** Hispanic or Latino**	136 (2.3)	53 (2.5)	71 (2.1)	12 (3.6)
** Not Hispanic or Latino**	4438 (75.7)	1484 (68.8)	2710 (80.3)	244 (73.9)
** Missing/unknown**	1289 (22.0)	620 (28.7)	595 (17.6)	74 (22.4)
**Practice types, *n* (%)**				
** Academic**	1119 (19.1)	383 (17.8)	655 (19.4)	81 (24.5)
** Community**	4585 (78.2)	1703 (79.0)	2640 (78.2)	242 (73.3)
** Both**	159 (2.7)	71 (3.3)	81 (2.4)	7 (2.1)
**Presence of brain metastasis^c^, *n* (%)**	630 (10.7)	251 (11.6)	334 (9.9)	45 (13.6)
**Presence of liver metastasis^c^, *n* (%)**	919 (15.7)	397 (18.4)	487 (14.4)	35 (10.6)
**History of smoking, *n* (%)**	5739 (97.9)	2112 (97.9)	3310 (98.0)	317 (96.1)
**ECOG performance score at index date**				
** 0**	1057 (18.0)	462 (21.4)	545 (16.1)	50 (15.2)
** 1**	2136 (36.4)	845 (39.2)	1174 (34.8)	117 (35.5)
** 2**	1070 (18.3)	417 (19.3)	587 (17.4)	66 (20.0)
** 3+**	337 (5.8)	120 (5.6)	198 (5.9)	19 (5.8)
** Missing/unknown**	1263 (21.5)	313 (14.5)	872 (25.8)	78 (23.6)
**Thoracic radiation^d^, *n* (%)**	232 (4.0)	70 (3.2)	151 (4.5)	11 (3.3)
**Year of 1L start date, *n* (%)**				
** <2018**	2535 (43.2)	0 (0.0)	2379 (70.5)	156 (47.3)
** ≥2018**	3328 (56.8)	2157 (100.0)	997 (29.5)	174 (52.7)

Abbreviations: 1L = first-line; ECOG = Eastern Cooperative Oncology Group.

a Patient cohort and analysis groups were indexed on the start date of 1L induction therapy.

b Other regimens included atezolizumab monotherapy (*N* = 47), carboplatin plus irinotecan (*N* = 37), topotecan monotherapy (*N* = 32), etc.

c Baseline period was defined as the time from 30 days prior to the ES-SCLC diagnosis date (inclusive) to the 1L induction therapy start date (inclusive).

d Thoracic radiation was investigated from 28 days prior to 7 days after the index date.

Baseline characteristics stratified by maintenance status are presented in Table S1. Overall, baseline characteristics were generally similar between the 1LM and non-maintenance cohorts, with the exception of a higher proportion of patients with ECOG performance score of 0-1 at 1L induction initiation in the 1LM cohort (64.9% vs 54.3%). In the 1LM cohort, baseline characteristics were similar between atezolizumab and durvalumab maintenance subgroups (Table S2).

### Treatment patterns over time

Through 2018, nearly all patients (93.8%) received 1L PE chemotherapy. The use of chemo-immunotherapy increased rapidly to 65.4% in 2019 and accounted for ∼80% of 1L induction therapy in 2022 and 2023 (Figure 2A). Among patients in the 1LM cohort (*N* = 1284), atezolizumab was the most common 1LM regimen from 2019 to 2023. The use of durvalumab increased from 8.8% of maintenance regimens in 2020 to 23.8% in 2023 (Figure 2B).

**Figure 2.**
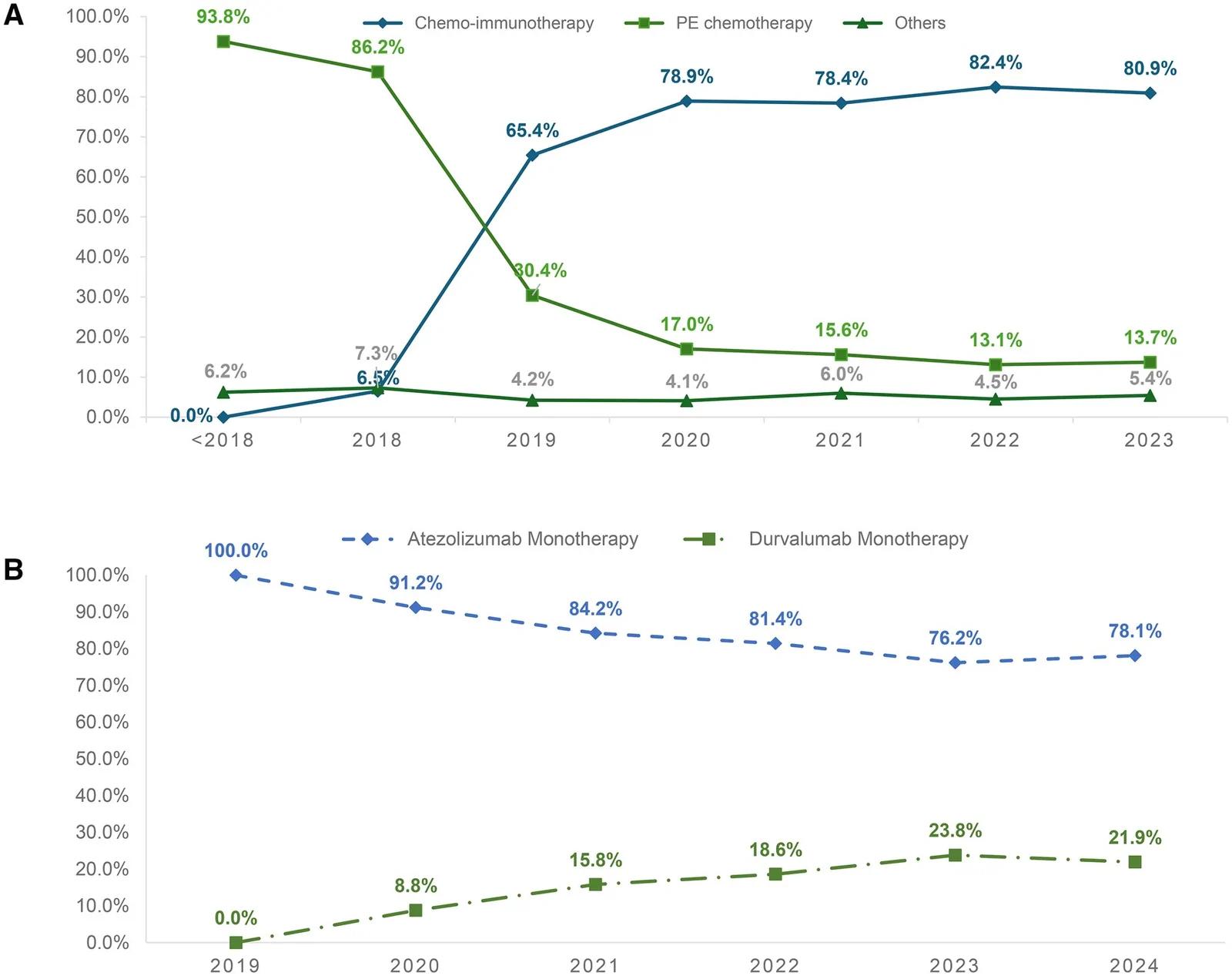
Treatment patterns of (A) first-line induction therapy over time in patients with extensive-stage small cell lung cancer, and (B) first-line maintenance therapy (atezolizumab or durvalumab) over time in patients with extensive-stage small cell lung cancer who previously received 1L chemo-immunotherapy. Two-panel figure showing changes in first-line treatment patterns over calendar time. Atezolizumab was approved by US FDA for the 1L treatment of ES-SCLC on March 18, 2019; durvalumab was approved by US FDA for the 1L treatment of ES-SCLC on March 20, 2020. Abbreviation: PE = platinum-based chemotherapy plus etoposide.

### Real-world survival outcomes from initiation of 1L therapy

The median rwOS and rwPFS from initiation of 1L therapy were 8.1 months (95% CI, 8.0-8.3) and 5.1 months (95% CI, 5.0-5.2), respectively. Median rwOS and rwPFS were numerically similar between patients receiving chemo-immunotherapy and those receiving PE chemotherapy in descriptive analyses (Table 2). However, patients treated with chemo-immunotherapy showed numerically higher rwOS rates at later follow-up timepoints compared with those receiving PE alone (12-month rwOS: 35.1% [95% CI, 33.1-37.3] vs 29.3% [95% CI, 27.7-30.9]; 24-month rwOS: 15.6% [95% CI, 14.0-17.4] vs 9.3% [95% CI, 8.3-10.4]).

**Table 2.** Real-world overall survival and real-world progression-free survival from initiation of first-line induction therapy among patients with extensive-stage small cell lung cancer.

	rwOS^a^					rwPFS^a^				
	*n*/*N*	Median, months (95% CI)	6-month, % (95% CI)	12-month, % (95% CI)	24-month, % (95% CI)	*n*/*N*	Median, months (95% CI)	6-month, % (95% CI)	12-month, % (95% CI)	24-month, % (95% CI)
**Overall 1L cohort**	5023/ 5861	8.1 (8.0-8.3)	65.3 (64.0-66.5)	31.2 (30.0-32.5)	11.8 (11.0-12.8)	4843/ 5655	5.1 (5.0-5.2)	38.3 (37.0-39.6)	8.3 (7.6-9.1)	3.5 (3.0-4.1)
**Patients in the overall 1L cohort by type of 1L induction therapy received**										
**Chemo-immunotherapy**	1751/ 2156	8.5 (8.1-8.9)	66.5 (64.5-68.6)	35.1 (33.1-37.3)	15.6 (14.0-17.4)	1873 /2096	5.2 (5.1-5.4)	37.7 (35.6-39.8)	10.1 (8.8-11.5)	5.3 (4.3-6.5)
**PE chemotherapy**	2999/ 3375	8.1 (7.9-8.3)	65.8 (64.2-67.5)	29.3 (27.7-30.9)	9.3 (8.3-10.4)	2726/ 3243	5.2 (5.1-5.3)	39.9 (38.1-41.7)	6.9 (6.0-7.9)	2.2 (1.7-2.8)
**Others**	273/ 330	6.1 (5.1-7.4)	51.2 (45.9-57.1)	25.9 (21.3-31.4)	15.0 (11.2-19.9)	244/ 316	2.9 (2.5-3.2)	26.5 (21.1-32.1)	9.7 (6.3-14.1)	3.8 (1.7-7.4)
**Patients in the chemo-immunotherapy cohort who did or did not receive 1L maintenance therapy (*N* = 2156)^b^**										
**Maintenance**	1004/ 1283	12.2 (11.4-13.1)	88.7 (87.0-90.5)	50.5 (47.8-53.4)	22.5 (20.1-25.1)	1156/ 1277	6.1 (5.9-6.3)	52.1 (49.3-54.9)	14.8 (12.9-16.9)	8.0 (6.5-9.7)
**No maintenance**	747/ 873	4.0 (3.7-4.3)	31.0 (27.9-34.4)	10.3 (8.3-12.7)	4.5 (3.1-6.5)	712/ 813	3.0 (2.8-3.1)	12.6 (10.2-15.2)	1.8 (0.9-3.1)	0.5 (0.1-1.5)
**Patients in the chemo-immunotherapy cohort with 1L maintenance therapy by type of 1L PD-L1 inhibitor receive^c^ (*N* = 1283)**										
**Atezolizumab**	863/ 1099	12.1 (11.2-13.1)	88.0 (85.9-89.8)	50.1 (47.1-53.1)	22.6 (19.9-25.3)	991/ 1094	6.1 (6.0-6.4)	52.5 (49.5-55.5)	14.9 (12.8-17.1)	8.1 (6.5-10.0)
**Durvalumab**	141/ 184	12.7 (10.9-14.4)	92.9 (88.1-95.8)	52.5 (44.9-59.5)	21.8 (15.6-28.7)	170/ 183	5.9 (5.7-6.4)	48.9 (41.5-55.9)	13.8 (9.2-19.2)	7.1 (3.8-11.7)

Abbreviations: 1L = first-line; PE = platinum-based chemotherapy plus etoposide; rwOS = real-world overall survival; rwPFS = real-world progression-free survival.

a Patients were excluded from the rwOS analysis if the death date only had year-level of granularity; patients who did not have a progression within the first line of therapy and had a death date anytime with only year-level granularity were excluded from the rwPFS analysis. Survival analyses were conducted among patients with sufficient data to derive rwOS and rwPFS; therefore, denominators vary across endpoints.

b The difference in cohort composition between the maintenance and non-maintenance groups limit comparisons due to potential selection and immortal time biases and should be interpreted with caution.

c Patients received the same PD-L1 inhibitors (atezolizumab or durvalumab) during both the induction therapy period and the subsequent maintenance phase.

Among patients treated with 1L chemo-immunotherapy, median rwOS was 12.2 months (95% CI, 11.4-13.1) in patients who subsequently met the operational maintenance therapy definition (∼60%; *n* = 1284 of 2157; maintenance cohort) and 4.0 months (95% CI, 3.7-4.3) in those who did not (Table 2). Corresponding, observed median rwPFS was 6.1 months (95% CI, 5.9-6.3) and 3.0 months (95% CI, 2.8-3.1), respectively (Table 2).

Median rwOS appeared broadly comparable between the atezolizumab and durvalumab maintenance subgroups. Median rwOS from initiation of 1L induction therapy was 12.1 months (95% CI, 11.2-13.1) and 12.7 months (95% CI, 10.9-14.4) in the atezolizumab and durvalumab subgroups, respectively (Table 2). The 12- and 24-month rwOS rates were also broadly comparable between the atezolizumab and durvalumab subgroups (12-month rwOS: 50.1% [95% CI, 47.1-53.1] vs 52.5% [95% CI, 44.9-59.5]; 24-month rwOS: 22.6% [95% CI, 19.9-25.3] vs 21.8% [95% CI, 15.6-28.7]). No meaningful differences in rwPFS were observed between the atezolizumab and durvalumab maintenance subgroups (Table 2).

### Real-world survival outcomes from initiation of 1L maintenance therapy

In the 1LM cohort, median rwOS was 8.8 months (95% CI, 8.3-9.7) and median rwPFS was 3.0 months (95% CI, 2.9-3.2) (Table 3). Median rwOS from the initiation of maintenance monotherapy was 8.7 months (95% CI, 8.0-9.8) and 9.3 months (95% CI, 8.0-11.5) in the atezolizumab and durvalumab subgroups, respectively (Table 3). Median rwPFS was 3.1 months (95% CI, 2.9-3.3) and 2.8 months (95% CI, 2.7-3.3), respectively (Table 3). Overall, no meaningful differences in rwOS or rwPFS were observed between atezolizumab and durvalumab in the maintenance setting.

**Table 3.** Real-world overall survival and real-world progression-free survival from initiation of first-line maintenance therapy among patients with extensive-stage small cell lung cancer.

Cohorts and analysis groups	rwOS^a^					rwPFS				
	*n*/*N*	Median, months (95% CI)	6-month, % (95% CI)	12-month, % (95% CI)	24-month, % (95% CI)	*n*/*N*	Median, months (95% CI)	6-month, % (95% CI)	12-month, % (95% CI)	24-month, % (95% CI)
**1LM cohort**	1004/ 1283	8.8 (8.3-9.7)	64.5 (61.9-67.2)	38.9 (36.2-41.7)	19.9 (17.6-22.5)	1145/ 1277	3.0 (2.9-3.2)	23.2 (20.9-25.6)	12.2 (10.4-14.1)	7.8 (6.3-9.5)
**Atezolizumab**	863/ 1099	8.7 (8.0-9.8)	63.9 (61.1-66.8)	38.8 (35.9-41.9)	19.9 (17.4-22.7)	978/ 1094	3.1 (2.9-3.3)	23.6 (21.1-26.2)	12.2 (10.3-14.3)	8.0 (6.4-9.9)
**Durvalumab**	141/ 184	9.3 (8.0-11.5)	68.0 (61.6-75.2)	39.4 (32.7-47.5)	19.8 (14.2-27.5)	167/ 183	2.8 (2.7-3.3)	20.9 (15.3-27.2)	12.1 (7.8-17.4)	6.17 (3.0-10.9)

Abbreviations: 1LM = first-line maintenance; rwOS = real-world overall survival; rwPFS = real-world progression-free survival.

a Patients were excluded from the rwOS analysis if the death date only had year-level of granularity; patients who did not have a progression within the first line of therapy and had a death date anytime with only year-level granularity were excluded from the rwPFS analysis. Survival analyses were conducted among patients with sufficient data to derive rwOS and rwPFS; therefore, denominators vary across endpoints.

### Subgroup analyses

Results of rwOS in prespecified subgroups are presented in Figure 3 for the overall 1L cohort and Figure 4 for the 1LM cohort. Corresponding rwPFS analyses are shown in Figures S1 and S2 (see online supplementary material for a color version of these figures).

**Figure 3.**
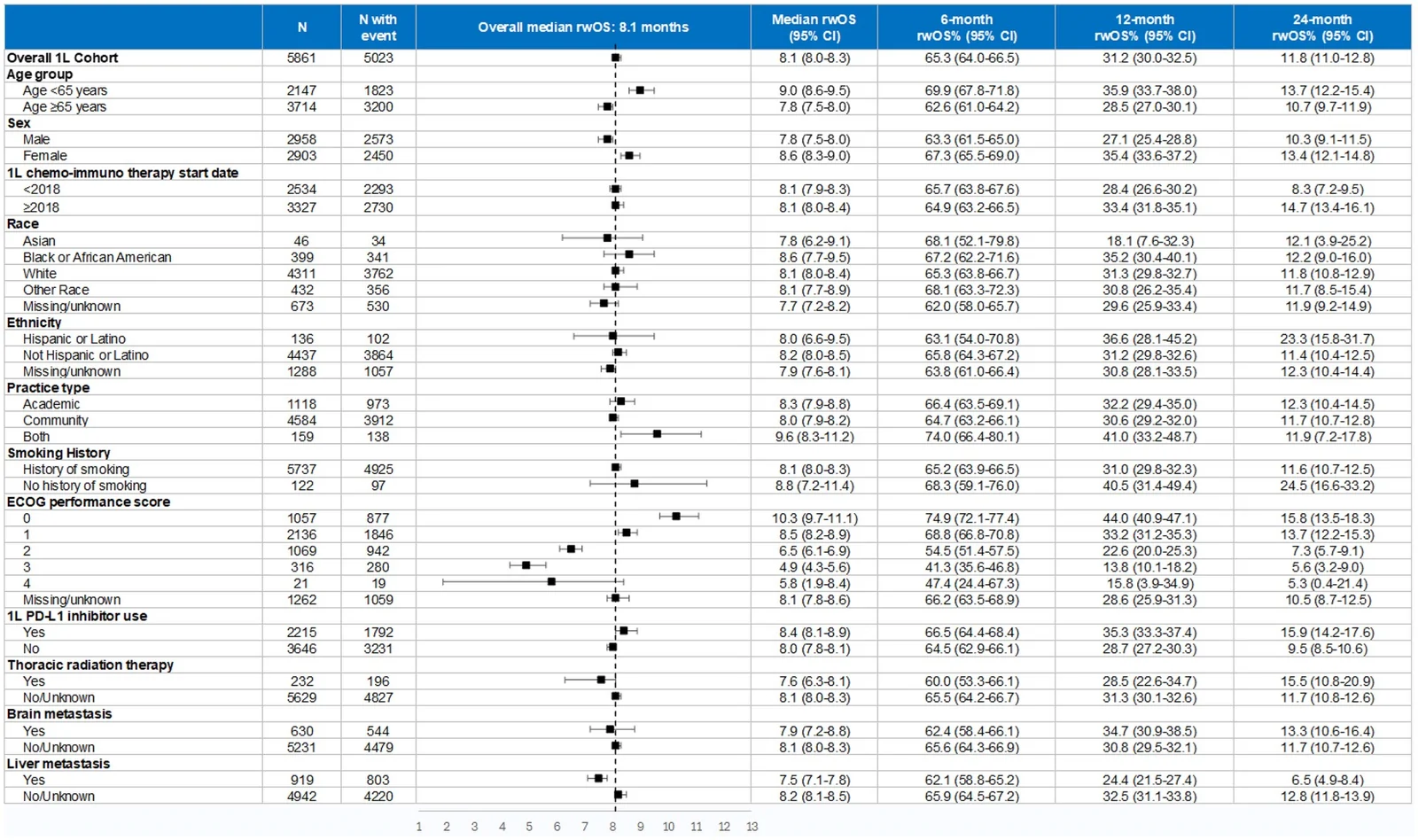
Real-world overall survival following initiation of first-line induction therapy by selected subgroups among the overall first-line cohort. Subgroup analysis of median real-world overall survival following initiation of first-line therapy among patients with ES-SCLC at initial diagnosis. Median survival estimates with 95% confidence intervals are displayed across prespecified subgroups. Abbreviations: 1L = first-line; ECOG = Eastern Cooperative Oncology Group; PD-L1 = programmed cell death ligand 1; rwOS = real-world overall survival.

**Figure 4.**
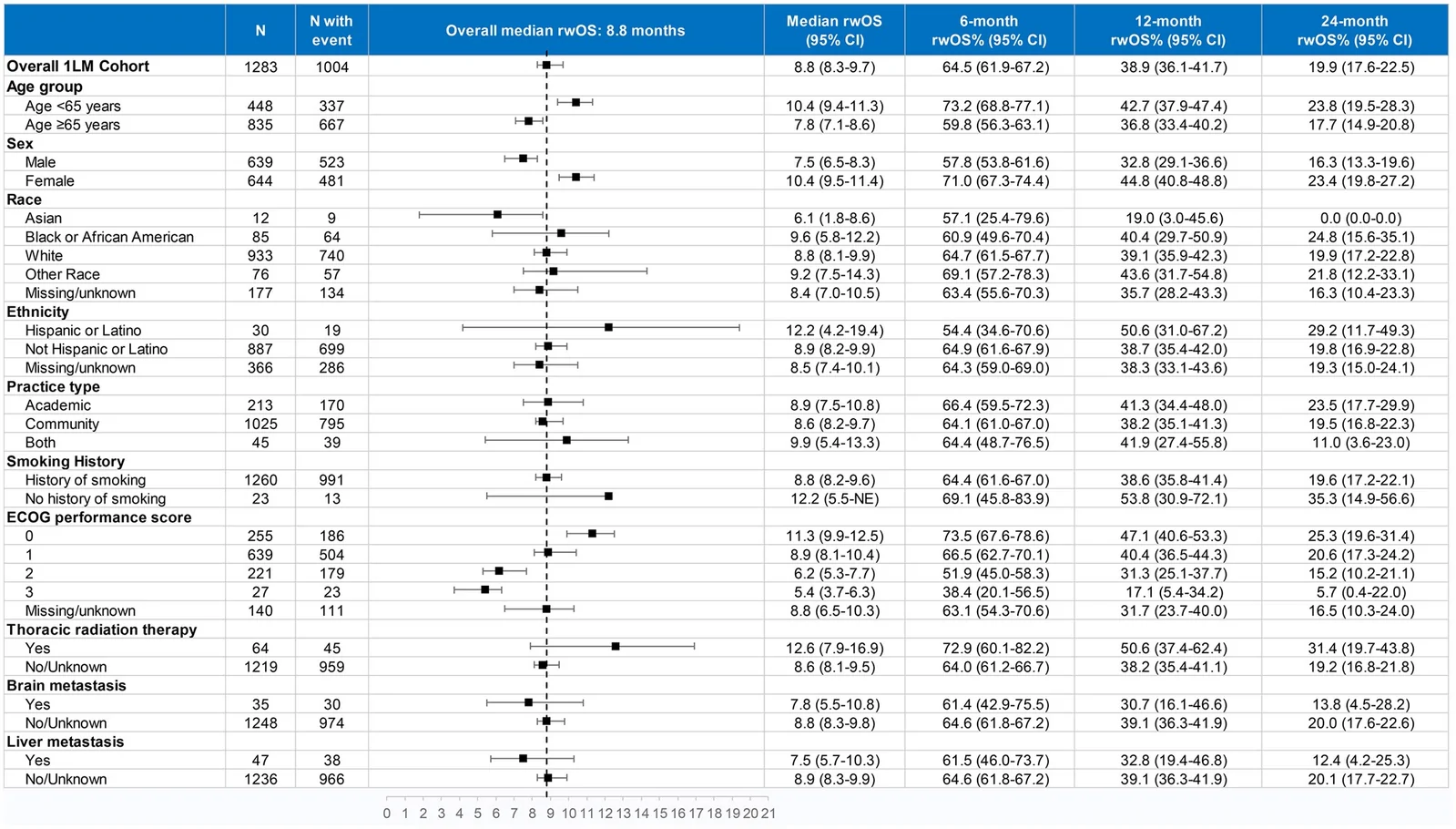
Real-world overall survival following initiation of first-line maintenance therapy by selected subgroups among the first-line maintenance cohort. Subgroup analysis of median real-world overall survival from initiation of first-line maintenance therapy among patients with ES-SCLC at initial diagnosis. Median survival estimates with 95% confidence intervals are displayed across prespecified subgroups. Abbreviations: 1LM = first-line maintenance; ECOG = Eastern Cooperative Oncology Group; rwOS = real-world overall survival.

In the 1LM cohort, subgroup analyses demonstrated longer median rwOS from initiation of maintenance therapy among patients age <65 years versus ≥65 years, women versus men, and those with ECOG performance score of 0-1 versus ≥2 (Figure 4). No meaningful differences in median rwPFS were observed across these subgroups, except among patients with brain metastasis, who had numerically shorter median rwPFS (Figure S2, see online supplementary material for a color version of this figure).

### Sensitivity analyses

Among 846 patients with ECOG performance score 0-1 who received ≥3 cycles of induction chemo-immunotherapy, median rwOS from initiation of 1L maintenance therapy was 9.9 months (95% CI, 8.6-10.7) and 9.9 months (95% CI, 7.9-12.2) in the atezolizumab and durvalumab subgroups, respectively (Table S3). At 12 months, rwOS rates were 41.8% (95% CI, 38.0-45.5) and 42.9% (95% CI, 34.2-51.3), respectively.

Sensitivity analyses using an alternate rwPFS definition, requiring radiographic documentation supporting the rwP event were conducted in the 1LM cohort without restriction on the number of induction cycles. Results were consistent with the primary analyses and showed no meaningful differences across maintenance subgroups (Table S4).

## Discussion

In this large, US-based real-world observational study of patients with ES-SCLC, we evaluated survival outcomes across both the 1L induction and maintenance phases of front-line treatment. By examining outcomes from initiation of induction chemo-immunotherapy as well as from the start of maintenance monotherapy, this analysis provides a comprehensive descriptive assessment of front-line real-world treatment patterns and outcomes.

Median rwOS (8.5 months; 95% CI, 8.1-8.9) observed in this study among patients receiving 1L chemo-immunotherapy was shorter than that reported in the IMpower133 (12.3 months; 95% CI, 10.8-15.8)^4^ and CASPIAN (12.9 months; 95% CI, 11.3-14.7)^5^ trials. These differences are expected and likely reflect well-established distinctions between randomized clinical trials and routine clinical practice. The IMpower133 and CASPIAN trial populations were largely restricted to patients with favorable ECOG performance scores of 0-1 and generally excluded individuals with uncontrolled or symptomatic central nervous system metastases, whereas our cohort included a broader and more clinically heterogenous population, including approximately 23% of patients with ECOG performance score ≥2 and 11% with documented brain metastases. In addition, real-world practice may involve greater variability in imaging frequency, treatment adherence, dose modifications, and supportive care, all of which may influence observed survival outcomes. Notably, sensitivity analyses restricted to patients with ECOG performance score 0-1 who received at least 3 cycles of induction chemo-immunotherapy yielded survival estimates (median rwOS: 9.9 months; 95% CI, 8.8-10.7) more closely aligned with those observed in clinical trials.^8^^,^^9^ These findings suggest that differences between trial efficacy and real-world effectiveness may be partially attributable to broader patient inclusion and differences in clinical eligibility encountered in routine oncology practice.^17–19^

Although median rwOS was numerically similar between chemo-immunotherapy and PE chemotherapy, the observed 12- and 24-month rwOS rates were numerically higher with chemo-immunotherapy. This pattern is directionally consistent with findings from IMpower133 and CASPIAN, in which the addition of PD-L1 inhibition was associated with improvements in landmark survival with longer follow-up.^4^^,^^5^ However, these descriptive, unadjusted analyses cannot establish a causal treatment effect or identify which patients may benefit.

Transition to maintenance therapy differed between clinical trials and our study. In IMpower133, approximately 77% of patients continued atezolizumab during the maintenance phase,^4^ whereas only about 60% of patients who initiated induction chemo-immunotherapy transitioned to maintenance therapy (≥2 doses of PD-L1 inhibitors) in our study. Differences in proportions may be attributable to differences in cohort composition and study design. Nevertheless, the lower transition rate observed in routine practice may reflect several factors such as clinical ineligibility, treatment related toxicity, patient and physician preferences and other factors, underscoring the importance of understanding attrition across lines of therapy in the real-world setting.

Patients who met the study specific maintenance therapy definition, had longer observed median rwOS than those who did not (12.2 months vs 4.0 months). However, this descriptive difference is inherently subject to selection and immortal time biases and should not be interpreted as causal. Initiation of maintenance therapy requires patients to remain progression-free and clinically stable following induction, thereby selecting for a more favorable prognosis. Furthermore, the non-maintenance cohort in this study included both patients who were not eligible for maintenance (eg, due to progression during induction or early discontinuation during induction) and those who were potentially eligible but did not initiate maintenance therapy. However, the specific reason for not initiating maintenance therapy could not be determined from the available data, and factors such as treatment intolerance, clinical deterioration, patient or clinician preference, or transitions in care could not be reliably determined. Accordingly, the maintenance and non-maintenance cohorts represent clinically selected populations and should not be directly compared as estimates of treatment effectiveness.

Consistent with prior observational studies, survival outcomes associated with atezolizumab- and durvalumab-based regimens in the 1L ES-SCLC setting, appeared broadly comparable in descriptive analyses.^10–13^ Several studies reported similar findings across different geographic settings,^10^^,^^13^ although some smaller, single-institution studies reported numerical differences between agents.^11^^,^^12^ These variations may reflect limited sample sizes, differences in patient populations, differences in clinical practice patterns, and residual confounding.

Importantly, our study extends prior work by separately evaluating survival from the start of maintenance monotherapy, an area not explored in previous real-world analyses. Median rwOS following maintenance initiation was shorter in our cohort than maintenance phase survival estimates reported in clinical trials. In IMpower133, median OS during the maintenance phase was 12.5 months (95% CI, 9.0-14.5)^8^ and in the IMforte trial, the atezolizumab monotherapy control arm demonstrated a median OS of 10.6 months (95% CI, 9.5-12.2).^9^ In contrast, median rwOS from maintenance start in our cohort was 8.7 months (95% CI, 8.0-9.8) with atezolizumab and 9.3 months (95% CI, 8.0-11.5) with durvalumab. This likely reflects the inclusion of patients with poorer performance status and more heterogenous disease characteristics in routine practice. Notably, maintenance phase survival outcomes in clinical trials for durvalumab have not been reported separately^20^ and our findings provide large-scale, real-world estimates for both agents in the maintenance setting.

The modest survival outcomes observed in the maintenance setting highlight an unmet need and provide context for emerging therapeutic strategies. Interim results from the phase 1b DeLLphi-303 study demonstrated comparable PFS between atezolizumab and durvalumab, each administered in combination with tarlatamab in the maintenance phase.^21^ Beyond DeLLphi-303, additional novel combinations and immunotherapeutic strategies are in development with the goal of improving outcomes of patients with ES-SCLC.^7^ As therapeutic options expand, improved patient selection may become increasingly important, and emerging molecular and transcriptomic classifications of SCLC may help identify patients most likely to benefit from immunotherapy-based treatments. Real-world evidence will remain essential to inform clinical decision making, particularly in populations underrepresented in clinical trials.

This study has limitations that should be considered when interpreting the findings. First, as a retrospective analysis of EHR-derived data, the results are dependent on the completeness and accuracy of routinely collected clinical documentation. ECOG performance status, an important prognostic factor, was missing for approximately 20% of patients, limiting our ability to fully contextualize survival outcomes for all patients included in the cohort. Second, identification of metastatic sites, including brain and liver metastases, relied on ICD codes. This may lead to under-ascertainment of metastases. Third, rwP events were abstracted from clinical documentation and thus may not be directly comparable to the events as determined in the context of a clinical trial using Response Evaluation Criteria in Solid Tumors (RECIST) criteria. The anatomical site and pattern of progression were unavailable. Sensitivity analyses using an alternative rwPFS definition based on radiologically documented progression events were conducted and yielded results consistent with the primary analysis. Fourth, the identification of maintenance therapy and transitions between lines of therapy were assigned using a clinician-informed, rule-based algorithm and may be misclassified. However, previously validated line-of-therapy algorithms based on clinical rationales were applied to enhance consistency in treatment classification. Fifth, our operational definition of maintenance therapy required at least 2 consecutive administrations. Although this was intended to distinguish an ongoing maintenance regimen from an isolated post-induction PD-L1 inhibitor administration, it may exclude some patients who clinically initiated maintenance but did not receive a second dose because of early progression, toxicity, clinical deterioration, or other reasons. However, available treatment-administration data alone could not reliably distinguish the clinical intent of an isolated post-induction administration. Sixth, specific reasons for treatment modification or failure to initiate maintenance were not reliably captured. Future studies should characterize reasons for attrition. Lastly, eligibility was restricted to ES-SCLC at initial diagnosis; findings may not generalize to patients receiving systemic therapy for recurrent or extensive-stage disease after an initial LS-SCLC diagnosis.

This study has several notable strengths. It leverages a large, contemporary, US-based EHR-derived dataset, enabling evaluation of treatment patterns and outcomes in a large geographically distributed population of patients with ES-SCLC receiving routine care across community and academic practices. Most patients (78.2%) were treated in community oncology practices, increasing the relevance of the findings to routine US community care. The analysis uniquely characterizes survival outcomes across both the first-line induction and maintenance phases, including evaluation from the initiation of maintenance therapy, which has been less well described in prior real-world studies. In addition, the inclusion of patients with a wider range of clinical characteristics, including those typically underrepresented in clinical trials, enhances the clinical relevance of the findings. The use of multiple sensitivity analyses further supports the robustness and consistency of the observed results.

Taken together, these findings highlight substantial attrition between induction chemo-immunotherapy and maintenance therapy in routine US oncology practice. Observed survival outcomes during the maintenance phase were broadly comparable between atezolizumab- and durvalumab-based regimens. These observations underscore the importance of better understanding factors influencing maintenance eligibility, treatment attrition, and outcomes in real-world settings, particularly as novel maintenance strategies continue to emerge in ES-SCLC.

## Conclusion

In this large, US-based real-world study of patients with ES-SCLC at initial diagnosis, we describe attrition patterns from 1L induction chemo-immunotherapy to maintenance. Observed survival associated with atezolizumab- and durvalumab-based regimens appeared broadly comparable when evaluated from both initiation of 1L induction chemo-immunotherapy and from the start of maintenance therapy. The poor real-world outcomes observed during 1L treatment highlight a continued need for strategies that improve disease control in ES-SCLC.

## Supplementary Material

oyag434_Supplementary_Data

## Data Availability

The data that support the findings of this study have been originated by Flatiron Health, Inc. Requests for data sharing by license or by permission for the specific purpose of replicating results in this manuscript can be submitted to PublicationsDataAccess@flatiron.com.
