# Supplementary material for: Real-world treatment patterns and survival outcomes in extensive-stage small cell lung cancer across first-line induction and maintenance therapy
Source: Oncologist. 2026 Nov 4;31(12):oyag434. doi: 10.1093/oncolo/oyag434 (PMC13750180; doi:10.1093/oncolo/oyag434)
Supplement: Supplementary file 1 [file oyag434_supplementary_data.zip › 1L_ES-SCLC_RWE_Supplement_Figures_The_Oncologist_(1).docx]

**Figure S1. Real-world progression-free survival following initiation of first-line induction therapy by selected subgroups among the overall first-line cohort.**
Abbreviations: 1L, first-line; CI, confidence interval; ECOG, Eastern Cooperative Oncology Group; PD-L1, programmed death ligand 1; rwPFS, real-world progression-free survival.

**Figure S2. Real-world progression-free survival following initiation of first-line maintenance therapy by selected subgroups among the first-line maintenance cohort.**

Abbreviations: 1LM, first-line maintenance; CI, confidence interval; ECOG, Eastern Cooperative Oncology Group; rwPFS, real-world progression-free survival.
