# Supplementary material for: Real-world treatment patterns and survival outcomes in extensive-stage small cell lung cancer across first-line induction and maintenance therapy
Source: Oncologist. 2026 Nov 4;31(12):oyag434. doi: 10.1093/oncolo/oyag434 (PMC13750180; doi:10.1093/oncolo/oyag434)
Supplement: Supplementary file 1 [file oyag434_supplementary_data.zip › Supplement_Data.zip!/Supplement_Data/1L ES-SCLC RWE_Supplement Tables_The Oncologist.docx]

**Table S1. Baseline characteristics of patients with extensive-stage small-cell lung cancer who did or did not receive first-line maintenance monotherapy^a^**

|  | 1L chemo-immunotherapy  (N=2,157) |  | 1L Maintenance Monotherapy | |
| --- | --- | --- | --- | --- |
|  |  |  | Yes (N=1,284) | No (N=873) |
| **Median age at index date, years (range)** | 68 (30-85) |  | 68 (30-85) | 69 (37-85) |
| **Age ≥65 years, n (%)** | 1,416 (65.6) |  | 823 (64.1) | 593 (67.9) |
| **Female, n (%)** | 1,098 (50.9) |  | 644 (50.2) | 454 (52.0) |
| **Race, n (%)** |  |  |  |  |
| Asian | 19 (0.9) |  | 12 (0.9) | 7 (0.8) |
| Black or African American | 151 (7.0) |  | 85 (6.6) | 66 (7.6) |
| White | 1,527 (70.8) |  | 934 (72.7) | 593 (67.9) |
| Other Race | 140 (6.5) |  | 76 (5.9) | 64 (7.3) |
| Missing/unknown | 320 (14.8) |  | 177 (13.8) | 143 (16.4) |
| **Ethnicity, n (%)** |  |  |  |  |
| Hispanic or Latino | 53 (2.5) |  | 30 (2.3) | 23 (2.6) |
| Not Hispanic or Latino | 1,484 (68.8) |  | 887 (69.1) | 597 (68.4) |
| Missing/unknown | 620 (28.7) |  | 367 (28.6) | 253 (29.0) |
| **Practice types, n (%)** |  |  |  |  |
| Academic | 383 (17.8) |  | 213 (16.6) | 170 (19.5) |
| Community | 1,703 (79.0) |  | 1,026 (79.9) | 677 (77.5) |
| Both | 71 (3.3) |  | 45 (3.5) | 26 (3.0) |
| **Presence of brain metastasis^b^, n (%)** | 251 (11.6) |  | 134 (10.4) | 117 (13.4) |
| **Presence of liver metastasis^b^, n (%)** | 397 (18.4) |  | 233 (18.1) | 164 (18.8) |
| **History of smoking, n (%)** | 2,112 (97.9) |  | 1,261 (98.2) | 851 (97.5) |
| **ECOG performance score at index date** |  |  |  |  |
| 0 | 462 (21.4) |  | 304 (23.7) | 158 (18.1) |
| 1 | 845 (39.2) |  | 529 (41.2) | 316 (36.2) |
| 2 | 417 (19.3) |  | 213 (16.6) | 204 (23.4) |
| 3 | 114 (5.3) |  | 51 (4.0) | 63 (7.2) |
| 4 | 6 (0.3) |  | 2 (0.2) | 4 (0.5) |
| Missing/unknown | 313 (14.5) |  | 185 (14.4) | 128 (14.7) |
| **Prior thoracic radiation^c^, n (%)** | 70 (3.2) |  | 47 (3.7) | 23 (2.6) |
| **Year of 1L start date, n (%)** |  |  |  |  |
| <2018 | 0 (0.0) |  | 0 (0.0) | 0 (0.0) |
| 2018 | 37 (1.7) |  | 17 (1.3) | 20 (2.3) |
| 2019 | 393 (18.2) |  | 260 (20.2) | 133 (15.2) |
| 2020 | 422 (19.6) |  | 248 (19.3) | 174 (19.9) |
| 2021 | 411 (19.1) |  | 252 (19.6) | 159 (18.2) |
| 2022 | 458 (21.2) |  | 257 (20.0) | 201 (23.0) |
| 2023 | 436 (20.2) |  | 250 (19.5) | 186 (21.3) |

a. Patient cohort and analysis groups were indexed on the start date of 1L induction therapy.
b. Baseline period was defined as the time from 30 days prior to the ES-SCLC diagnosis date (inclusive) to the 1L combination therapy start date (inclusive).
c. Prior refers to -28 to +7-day of the index date.
Abbreviations: 1L, first-line; ECOG, Eastern Cooperative Oncology Group.

**Table S2. Baseline characteristics of patients with extensive-stage small-cell lung cancer who received first-line maintenance monotherapy^a^**

|  | 1L Maintenance Cohort  (N=1,284) |  | 1L Maintenance Monotherapy | |
| --- | --- | --- | --- | --- |
|  |  |  | Atezolizumab (N=1,100) | Durvalumab (N=184) |
| **Median age at index date, years (range)** | 68 (30-85) |  | 68 (30-85) | 68 (40-85) |
| **Age ≥65 years, n (%)** | 835 (65.0) |  | 713 (64.8) | 122 (66.3) |
| **Female, n (%)** | 644 (50.2) |  | 549 (49.9) | 95 (51.6) |
| **Race, n (%)** |  |  |  |  |
| Asian | 12 (0.9) |  | 10 (0.9) | ≤5 |
| Black or African American | 85 (6.6) |  | 81 (7.4) | ≤5 |
| White | 934 (72.7) |  | 796 (72.4) | 138 (75.0) |
| Other Race | 76 (5.9) |  | 64 (5.8) | 12 (6.5) |
| Missing/unknown | 177 (13.8) |  | 149 (13.5) | 28 (15.2) |
| **Ethnicity, n (%)** |  |  |  |  |
| Hispanic or Latino | 30 (2.3) |  | 23 (2.1) | 7 (3.8) |
| Not Hispanic or Latino | 887 (69.1) |  | 770 (70.0) | 117 (63.6) |
| Missing/unknown | 367 (28.6) |  | 307 (27.9) | 60 (32.6) |
| **Practice types, n (%)** |  |  |  |  |
| Academic | 213 (16.6) |  | 184 (16.7) | 29 (15.8) |
| Community | 1,026 (79.9) |  | 879 (79.9) | 147 (79.9) |
| Both | 45 (3.5) |  | 37 (3.4) | 8 (4.3) |
| **Presence of brain metastasis^b^, n (%)** | 206 (16.0) |  | 172 (15.6) | 34 (18.5) |
| **Presence of liver metastasis^b^, n (%)** | 298 (23.2) |  | 241 (21.9) | 57 (31.0) |
| **History of smoking, n (%)** | 1,261 (98.2) |  | 1,079 (98.1) | 182 (98.9) |
| **ECOG performance score at index date** |  |  |  |  |
| 0 | 255 (19.9) |  | 214 (19.5) | 41 (22.3) |
| 1 | 639 (49.8) |  | 540 (49.1) | 99 (53.8) |
| 2 | 222 (17.3) |  | 193 (17.5) | 29 (15.8) |
| 3+ | 28 (2.2) |  | 24 (2.2) | 4 (2.2) |
| Missing/unknown | 140 (10.9) |  | 129 (11.7) | 11 (6.0) |
| **Prior thoracic radiation^c^, n (%)** | 64 (5.0) |  | 58 (5.3) | 6 (3.3) |
| **Year of 1LM start date, n (%)** |  |  |  |  |
| ≤2018 | 0 (0.0) |  | 0 (0.0) | 0 (0.0) |
| 2019 | 205 (16.0) |  | 205 (18.6) | 0 (0.0) |
| 2020 | 260 (20.2) |  | 237 (21.5) | 23 (12.5) |
| 2021 | 241 (18.8) |  | 203 (18.5) | 38 (20.7) |
| 2022 | 253 (19.7) |  | 206 (18.7) | 47 (25.5) |
| 2023 | 261 (20.3) |  | 199 (18.1) | 62 (33.7) |
| 2024 | 64 (5.0) |  | 50 (4.5) | 14 (7.6) |

a. Patient cohort and analysis groups were indexed on the start date of 1L maintenance therapy.
b. Baseline period was defined as the time from 30 days prior to the ES-SCLC diagnosis date (inclusive) to the 1L maintenance therapy start date (inclusive).
c. Prior refers to -28 to +7-day of the index date.
Abbreviations: 1L, first-line; 1LM, first-line maintenance; ECOG, Eastern Cooperative Oncology Group.

**Table S3. Real-world overall survival and real-world progression-free survival from initiation of first-line maintenance therapy among patients with extensive-stage small-cell lung cancer who had Eastern Cooperative Oncology Group performance score 0–1 and received ≥3 cycles of induction chemo-immunotherapy**

| Cohorts and analysis groups | rwOS^a^ | | | | |  | rwPFS^a^ | | | | |
| --- | --- | --- | --- | --- | --- | --- | --- | --- | --- | --- | --- |
|  | n/N | Median, months (95% CI) | 6-month % (95% CI) | 12-month % (95% CI) | 24-month % (95% CI) |  | n/N | Median, months (95% CI) | 6-month % (95% CI) | 12-month % (95% CI) | 24-month % (95% CI) |
| **1LM cohort** | 660/846 | 9.9  (8.8-10.7) | 68.4  (65.3-71.5) | 41.9  (38.5-45.4) | 21.5  (18.5-24.6) |  | 755/844 | 3.1  (2.9-3.3) | 23.7  (20.8-26.7) | 12.8  (10.6-15.2) | 8.2  (6.3-10.3) |
| **Atezolizumab** | 558/712 | 9.9  (8.6-10.7) | 68.14 (64.5-71.5) | 41.8 (38.0-45.5) | 21.7  (18.5-25.1) |  | 634/710 | 3.1  (2.9-3.3) | 23.7  (20.6-26.9) | 12.8  (10.4-15.5) | 8.2  (6.2-10.6) |
| **Durvalumab** | 102/134 | 9.9  (7.9-12.2) | 70.1  (61.5-77.1) | 42.9  (34.2-51.3) | 20.2  (13.0-28.5) |  | 121/134 | 2.9  (2.7-3.7) | 23.8  (16.9-31.4) | 12.7  (7.6-19.1) | 7.9  (3.9-13.7) |

a. Patients were excluded from the rwOS analysis if the death date only had year-level of granularity; patients who did not have a progression within the first line of therapy and had a death date anytime with only year-level granularity were excluded from the rwPFS analysis.

Abbreviations: 1LM, first-line maintenance; CI, confidence interval; rwOS, real-world overall survival; rwPFS, real-world progression-free survival.

**Table S4. Real-world progression-free survival from initiation of first-line maintenance therapy based on real-world progression events after a radiographic scan**

| Cohorts and analysis groups | rwPFS^a^ | | | | |
| --- | --- | --- | --- | --- | --- |
|  | n/N | Median, months (95% CI) | 6-month % (95% CI) | 12-month % (95% CI) | 24-month % (95% CI) |
| **1LM cohort** | 1141/1277 | 3.0  (2.9-3.2) | 23.3  (21.0-25.7) | 12.2  (10.4-14.2) | 8.0  (6.5-9.7) |
| **Atezolizumab** | 976/1094 | 3.1  (2.9-3.3) | 23.7  (21.2-26.3) | 12.2  (10.3-14.3) | 8.1  (6.4-9.9) |
| **Durvalumab** | 165/183 | 2.8  (2.7-3.4) | 21.1  (15.4-27.4) | 12.2  (7.8-17.6) | 7.3  (3.9-12.0) |

a. Patients who did not have a progression within the first line of therapy and had a death date anytime with only year-level granularity were excluded from the rwPFS analysis.

Abbreviations: 1LM, first-line maintenance; CI, confidence interval; rwPFS, real-world progression-free survival; rwP, real-world progression.
